# Analysis on the Effects of the Human Body on the Performance of Electro-Textile Antennas for Wearable Monitoring and Tracking Application

**DOI:** 10.3390/ma12101636

**Published:** 2019-05-19

**Authors:** Nurul Huda Abd Rahman, Yoshihide Yamada, Muhammad Shakir Amin Nordin

**Affiliations:** 1 Malaysia-Japan International Institute of Technology, Universiti Teknologi Malaysia, Jalan Sultan Yahya Petra, Kuala Lumpur 54100, Malaysia; ndayamada@yahoo.co.jp; 2 Faculty of Electrical Engineering, Universiti Teknologi MARA, Shah Alam 40450, Selangor, Malaysia; shakiraminnordin@gmail.com

**Keywords:** wearable devices, on-body antenna, wireless body area network, wireless sensing, smart material, wearable antenna

## Abstract

Previous works have shown that wearable antennas can operate ideally in free space; however, degradation in performance, specifically in terms of frequency shifts and efficiency was observed when an antenna structure was in close proximity to the human body. These issues have been highlighted many times yet, systematic and numerical analysis on how the dielectric characteristics may affect the technical behavior of the antenna has not been discussed in detail. In this paper, a wearable antenna, developed from a new electro-textile material has been designed, and the step-by-step manufacturing process is presented. Through analysis of the frequency detuning effect, the on-body behavior of the antenna is evaluated by focusing on quantifying the changes of its input impedance and near-field distribution caused by the presence of lossy dielectric material. When the antenna is attached to the top of the body fat phantom, there is an increase of 17% in impedance, followed by 19% for the muscle phantom and 20% for the blood phantom. These phenomena correlate with the electric field intensities (V/m) observed closely at the antenna through various layers of mediums (*z*-axis) and along antenna edges (*y*-axis), which have shown significant increments of 29.7% in fat, 35.3% in muscle and 36.1% in blood as compared to free space. This scenario has consequently shown that a significant amount of energy is absorbed in the phantoms instead of radiated to the air which has caused a substantial drop in efficiency and gain. Performance verification is also demonstrated by using a fabricated human muscle phantom, with a dielectric constant of 48, loss tangent of 0.29 and conductivity of 1.22 S/m.

## 1. Introduction

**Figure 1:**
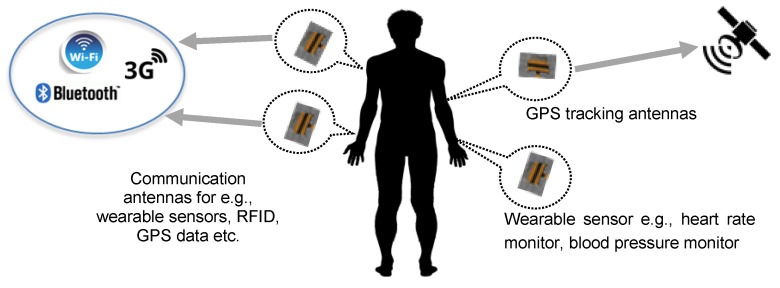
Examples of wearable antennas integrated with wearable electronic devices for many applications. Through wearable technology, various Internet of Things (IoT) applications can be supported such as healthcare monitoring via wearable sensors, human tracking through body-centric radio-frequency identification (RFID), sportsmen or patient tracking via a wearable global positioning system (GPS) receiver and many more.

Wearable antennas are highly in demand to support various wearable technologies due to the advantage of being flexible, lightweight and easily integrated with garments. Through wearable technology, a wireless body sensing system is now able to support various Internet of Things (IoT) applications such as for high speed communication, tracking, health monitoring and radio-frequency identification (RFID) [1,2,3], as illustrated in Figure 1. In wearable devices, antenna is important to support many wireless communication aspects such as for in-body communication, on-body communication and off-body communication [4]. Many considerations should be taken into account when designing a wearable antenna such as interconnectivity issues, consistency of performance and comfortability of users [5,6,7,8]. Due to the importance of these issues in antenna designing, this paper proposed a customized electro-textile fabric, and its performance consistency is analyzed with respect to on-body factors. 

**Figure 2:**
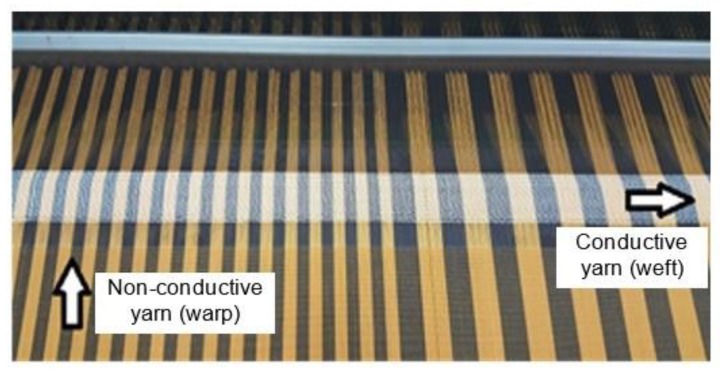
Warp and weft of the manufactured electro-textile fabric. The conducting part was made from a mixture of copper and polyester threads, with the weight ratio of 83% and 17% for copper and polyester, respectively.

Conventional textile-based wearable antennas are normally fabricated on regular fabrics, and integrated with copper-based radiating materials such as copper foil, copper thread, copper tape or copper powder [9]. For these structures, antenna substrate is normally represented by non-conductive material such as cotton, polyester or woven, and the integration of copper as the radiating element is done through various techniques such as embroidery, manual sewing, ironing, gluing etc. [10]. The main drawback of the conventional or off-the-shelf conductive fabric is the sustainability issue, where the antenna can be easily detached from the fabric after being washed or worn for many times [11]. In this research, a customized and more structurally practical conductive fabric as shown in Figure 2 was developed and analyzed. The electrical characteristics of this fabric have been determined through transmission line method; [12] however, the on-body performance has yet to be verified. 

In practice, wearable antennas are designed to be integrated with wearable devices and to be worn on the human body. Thus, the effects of the human body on antenna technical performance are significant and must be considered [4,13,14]. Previously, detuning effects were observed when an antenna was in close proximity to the human body; however, quantitative analysis in relation to impedance change and near-field distribution was not shown [15]. In order to minimize the detuning effects, a slotted planar inverted-F antenna (PIFA) was proposed [16] with a very broad bandwidth as the main feature, but in that particular work, measurement with the human body was not conducted. Numerical analysis of input impedance and current distribution with respect to the human body’s presence were performed theoretically by [17] using Green’s function in method of moment (MoM) code. Nevertheless, the analysis was limited to dipole antenna, and not specifically validated for textile-based materials. Similarly, as shown by [18], the effect of antenna-body-separation distance was presented, yet, the impedance change was not discussed numerically in terms of its magnitude or trends. Furthermore, the antennas used were not based on either planar structure or full-textile configuration, similar to the works done by [19,20,21]. The analysis of in-body and on-body antenna through in-pig measurement setup has been demonstrated in [4], but the focus of the paper was on miniaturization of the antenna design, which was not restricted to textile-based materials thus, low-loss robust substrate e.g., RO5880 was used. The most related work was shown in [22], where the comparison of textile antenna efficiency and gain were done with respect to power losses and field intensity inside the body. However, as concluded by the authors, there were some inconsistencies of test environment that should be taken into account when performing the measurement e.g., the antenna should be positioned on the same human body at the exact same location. Therefore, as proposed in this article, in order to reduce the uncertainties, a model of human body parts must be fabricated with respect to the actual dielectric properties to ensure a consistent test environment. Hence, systematic and quantitative analysis can be done accurately. Several researchers have done on-body analysis by using human phantoms but the main subjects of their works were different, and mainly focused on ways to enhance the antenna performance such as miniaturizing the structure, improving the gain or reducing the specific absorption rate (SAR) of the antenna [23].

In this paper, detailed analysis was carried out by studying the antenna characteristics based on phantoms of human body parts with various dielectric constants, *ε_r_* and conductivity, *σ* depending on the actual dielectric properties of the human body parts. Here, the behavior of electric field and magnetic surface current that penetrates through a high-loss medium such as a human phantom were demonstrated and numerically analyzed in terms of the near-field magnitude. To demonstrate the effects of on-body condition to the electro-textile (e-textile) antenna performance, a wearable antenna which was designed at 1.575 GHz frequency, was used. The frequency was chosen based on the allocated spectrum for GPS tracking devices, which can be implemented for many purposes such as patient or pet-tracking, health monitoring or locating military personnel during search and rescue missions. All simulation works were done by using a comprehensive 3D electromagnetic software called CST, and verification of results was done through bench and anechoic chamber measurements. A gel-based human muscle phantom was fabricated with *ε_r_* and *σ* similar to human muscle tissue to demonstrate the on-body behavior. Human muscle was chosen because the conductivity σ and dielectric constant *ε_r_* were considered as average, as compared to other human body parts such as fat (lowest *ε_r_*) and blood (highest *ε_r_*). Furthermore, it makes up the largest proportion of the human arms.

## 2. Methods and Processes 

**Figure 3:**
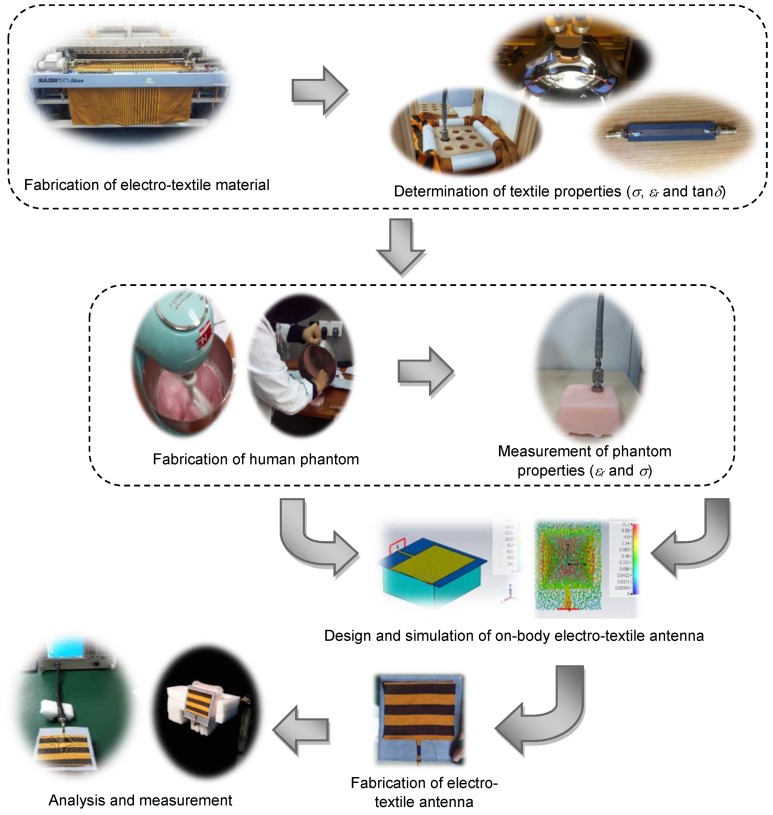
A flowchart showing the overall process of fabrication, characterization, design, simulation, measurement and analysis of the electro-textile antenna performance in on-body conditions.

Figure 3 shows the overall flow of the work, starting with fabrication and characterization of electro-textile materials, as done in [12]. Then, human muscle phantom was fabricated and the dielectric properties such as loss tangent (tan*δ*), dielectric constant (*ε_r_*) and conductivity (*σ*) were measured. The measured and calculated parameters were used in the design and simulation phase to ensure optimum antenna performance in ideal conditions. To verify the performance, an electro- textile antenna was fabricated based on its optimum design in ideal conditions. Measurement was conducted and important parameters such as return loss (*S_11_*), impedance (*Z_in_*) and radiation pattern were recorded and analyzed. Based on the experimental results, numerical data were analyzed in depth based on the following scenarios; ideal (free space) and on-body (with a human phantom, for various phantom properties). By observing the near-field data (field distribution) and surface current with respect to impedance change, clear correlation and evidence of a significant change to antenna performance were shown. These findings may contribute towards more accurate wearable antenna designing in the future. 

## 3. Characterization, Design and Fabrication of Wearable Antenna

In this section, the design concept of the basic planar rectangular electro-textile antenna is shown. There are three sub-sections; production process of e-textile, determination of dielectric and electrical properties, and design of e-textile antenna.

### 3.1. Electro-Textile Production

**Figure 4:**
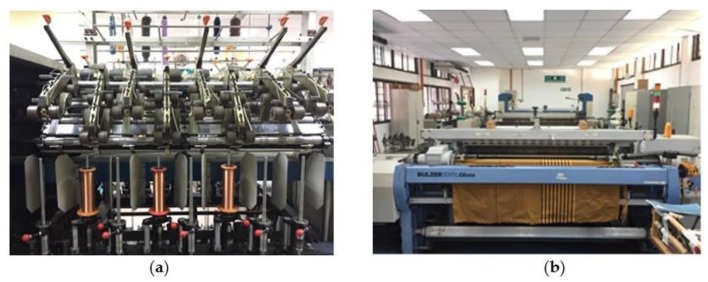
Electro-textile production process: (**a**) Spindle-spinning process for yarn formation; (**b**) Weaving process for e-textile formation.

Electro-textile was manufactured through integration of conductive and non-conductive yarns. In this project, conductive yarns were constructed first by twisting copper threads (0.14mm in diameter) with polyester threads by using a hollow-spindle machine as shown in Figure 4a. After that, the process continued with the production of e-textile fabric by using the ‘plain’ weaving technique. In the weaving process, Sulzer Textil G6300 rapier weaving machine (Sulzer, Winterthur, Switzerland) as shown in Figure 4b was used. The manufactured e-textile fabric consists of 25 wefts/inch in a horizontal direction and 80 warps/inch in a vertical direction, which was selected based on the optimum setting and the machine’s limitations. In this fabric construction, conductive yarns were used as the wefts, whilst non-conductive threads (polyester) were in the warp direction as shown previously in Figure 2. 

### 3.2. Electrical and Dielectric Properties

There are two elements in the design of textile antenna; conductive textile as the radiating element and non-conductive textile as the non-radiating (substrate) element. For the radiating element, electrical conductivity (*σ*) is very important, whereas, for the substrate, dielectric properties such as dielectric constant (*ε_r_*) and loss tangent (tan*δ*) play significant roles. Table 1 shows the properties of electro-textile fabricated in this paper. Electrical conductivity (*σ*) and the fabric’s thickness (*t*) are two important parameters that shall influence the antenna performance. The *σ* was calculated from resistance (*R*) value, obtained through current-voltage (I-V) probe method [24] and verified through propagation constant (*γ*) parameter obtained from the newly proposed strip line measurement technique [12]. The technique was developed based on the two-port transmission line, and the *σ* was derived using Equation (1), whereby *ε* is the material’s permittivity and *µ* is the material’s permeability:

(1) $$\sigma =\frac{\omega^{3}\varepsilon^{2}\mu }{\alpha^{2}h^{2}(2\alpha^{2}+2\omega^{2}\varepsilon \mu )}$$

**Figure 5:**
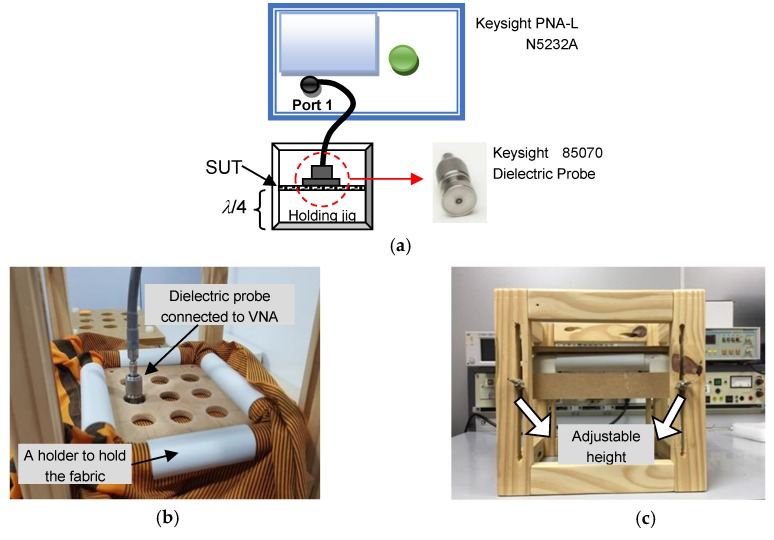
Measurement of dielectric properties: (**a**) Equipment arrangement which consists of dielectric probe, network analyzer and holding jig; (**b**) Customized jig with holes and holders to ensure consistent readings and to control fabric stiffness; (**c**) Adjustable height feature to allow measurement of many samples at various operational frequencies. VNA, vector network analyzer; SUT, sample-under-test.

Although the main component in the e-textile was copper, the calculated *σ* was lower than the *σ* of pure copper due to the presence of non-conductive material such as polyester inside the conductive thread. This has resulted in a less ideal performance of the textile-based antenna as compared to the full-metallic antenna, which will be discussed later in this paper. The composition of fabric was calculated based on mass per unit area, according to ASTM D3776 [25]. In this research, polyester fabric having low *ε_r_* and moderate tan*δ* was used as the substrate. This polyester material was chosen due to its suitability and comfortability features to be used for daily wear. The dielectric characteristics of the polyester substrate were determined prior to performing antenna design and simulation to ensure accurate analysis. Both *ε_r_* and tan*δ* were calculated through the coaxial probe measurement technique by using the Keysight 85070 dielectric probe kit (Keysight Technologies, Santa Rosa, CA, USA), which consists of a vector network analyzer (VNA), a coaxial probe and software to display the complex permittivity data obtained through *S*-parameter conversion, measured by the network analyzer [26]. The measurement setup is illustrated in Figure 5a. A customized jig with multiple holes as shown in Figure 5b was developed to allow the fabric sample to be stretched uniformly on the surface to ensure consistent and accurate readings. Furthermore, the height of fabric sample from the table’s surface must be more than *λ*/4 to avoid multiple reflection, hence, an additional feature as shown in Figure 5c was included in this jig in which the height of sample can be adjusted according to its operational frequency, *f_o_*.

In the *ε_r_* measurement, the *ε*^’^ (known as the real part of the *ε_r_*) and the *ε*^”^ (known as the imaginary part of the *ε_r_*) were measured by contacting the dielectric probe to the flat surface of the sample-under-test (SUT). The complex permittivity is defined as follows:

(2) $$\varepsilon_{r}=\varepsilon^{\prime }-j\varepsilon^{"}$$

where the *ε*^’^ and *ε*^”^ reflect the characteristics of a lossy material in terms of its tan*δ*, as shown by Equation (3) [26]:

(3) $$\tan\delta =\frac{\varepsilon^{"}}{\varepsilon^{\prime }}$$

Based on the measurement at 1.575 GHz, the polyester substrate had a *ε_r_* of 1.36 and a tan*δ* of 0.031, with a thickness, *h* of 1.4 mm. 

### 3.3. Antenna Design (Ideal and On-Body Conditions)

**Figure 6:**
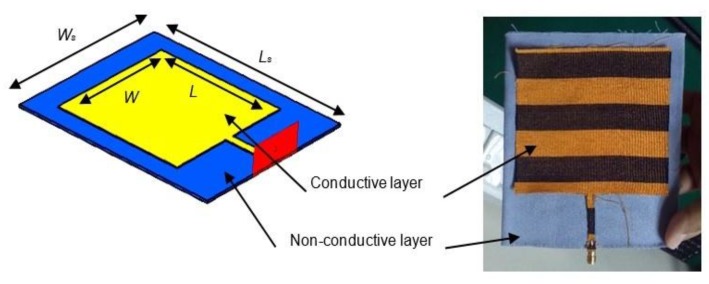
Geometry and structure of 1.575 GHz rectangular electro-textile antenna in the free space condition (Case A), with W/L ratio of 1.1 for the conductive layer and W_s_ /L_s_ ratio of 0.9 for the non-conductive layer. These values have been optimized based on the free space condition.

For analysis, a rectangular antenna with an edge-fed transmission line was chosen. This antenna was chosen due to the symmetrical geometry, which makes it easier to design, fabricate and analyze. The performance of the designed antenna should be improved further; however, in this paper, the focus is on studying and understanding the technical behavior of the antenna when located near the human body. Thus, based on the electrical properties of conductive material and the dielectric characteristics of the polyester substrate, a 1.575 GHz electro-textile antenna was designed. The antenna consists of a rectangular radiating element with a full ground plane, made by using the fabricated conductive textile material. The geometry and fabricated structure of the electro-textile antenna is shown in Figure 6. The integration of the top radiator (conductive textile) and the bottom ground plane to the dielectric layer (polyester) was performed by using a fabric glue that was heated up by using a hot-compress machine during assembly to ensure strong attachment of fabric layers.

**Figure 7:**
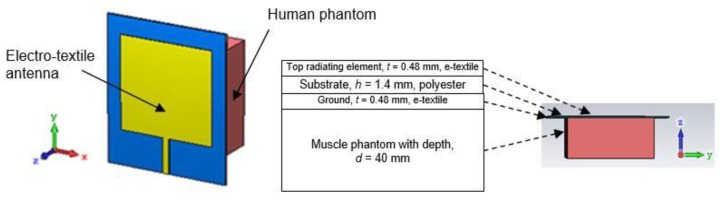
Configuration for on-body condition analysis (Case B). During simulation, the antenna is attached to the human phantom with various dielectric constant, *ε_r_* and conductivity, *σ* to represent the exact properties of human body fat, muscle and blood.

In wearable GPS tracking devices, electro-textile antenna should operate on the human body and be specifically placed at any body locations such as the arm, wrist, shoulder or leg. For that reason, the effects must be extrapolated and analyzed. In order to understand the impact of on-body condition to a wearable antenna, two situations were simulated, demonstrated and studied in this article:

1. Case A: Antenna in free space (without phantom)—Figure 6.
2. Case B: Antenna on a human phantom—Figure 7.

For Case B, a rectangular shaped dielectric material (phantom) that has similar properties with the corresponding human body part was attached as another structure to the ideal antenna. Here, important properties of body tissues which are *ε_r_* and *σ* were set. For this simulation, the *ε_r_* and *σ* at 1.575 GHz were varied for three cases; body fat (*ε_r_* = 12, *σ* = 0.17 S/m), muscle (*ε_r_* = 48, *σ* = 1.22 S/m) and blood (*ε_r_* = 64, *σ* = 1.9 S/m) [27].

## 4. Fabrication of Human Muscle Phantom

**Figure 8:**
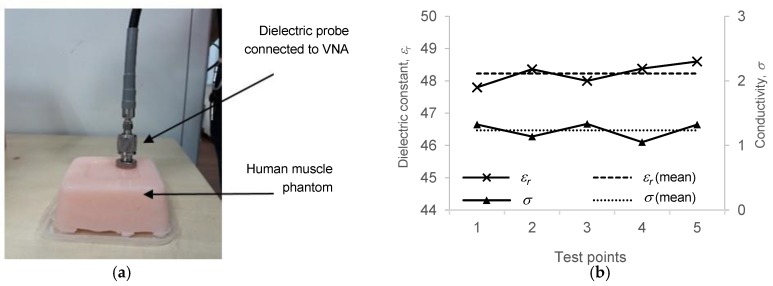
(**a**) Measurement of dielectric properties of the muscle phantom. The readings were taken at five different locations on the phantom’s surface to ensure the accuracy of the data; (**b**) Graph showing the measured *ε_r_* and *σ*, and comparison with the mean values.

A human phantom is a physical model having similar properties as human biological tissues [28]. For antenna performance validation, measurement of scattering parameters (*S_11_*) for on-body condition can be done by using a real human body part; however, due to the complexity of antenna three-dimensional (3D) radiation pattern measurement, phantom fabrication was proposed to represent a human-like material. The 3D radiation pattern measurement for on-body wearable antenna must be conducted inside an anechoic chamber. Due to radiation, health concerns and mounting complexity, it is not advisable for a real human body to be included as part of the measurement setup. In this paper, in order to validate the analysis, human phantom fabrication was simplified to human muscle which makes up the largest proportion of the human arm. Thus, based on the specified parameters, a suitable mixing ratio of chemicals was suggested. Fabrication was done several times to ensure that the required dielectric properties were achieved. Finally, after several trials, a good muscle phantom was developed by using the chemical composition as shown in Table 2. After the fabrication, the *ε_r_* of the phantom was determined by using the coaxial probe technique as shown in Figure 8a. The data from five measurements were taken and the average *ε_r_* and *σ* were calculated, as shown in Figure 8b. The results are as follows; *ε_r_* = 48.2, *σ* = 1.23 and tan*δ* = 0.292. There were only slight variations between the measured data as compared to the theoretical values, with the calculated standard deviation (*σ_x_*) of 0.3 for *ε_r_*, 0.12 for *σ* and 0.03 for tan*δ*. 

## 5. Results

**Figure 9:**
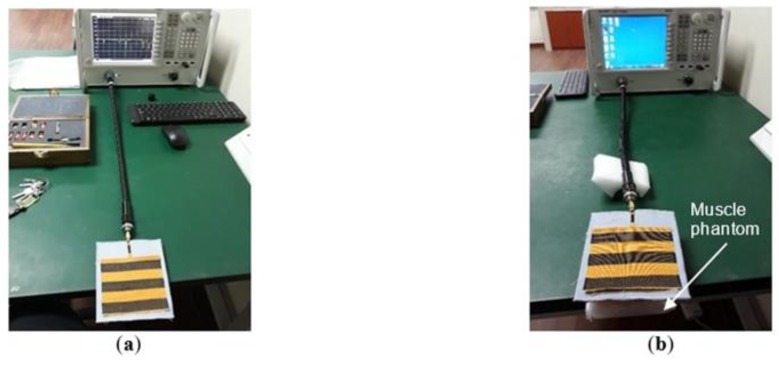
Measurement setup for return loss (*S_11_*): (**a**) In free space (Case A); (**b**) In the presence of the muscle phantom (Case B).

**Figure 10:**
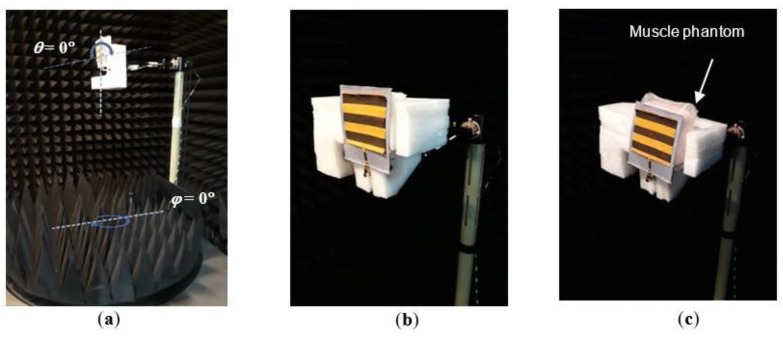
(**a**) Measurement configuration for the radiation pattern, showing the starting point of *θ* and *ϕ*; (**b**) Antenna configuration in free space (Case A); (**c**) Antenna configuration in the presence of the muscle phantom (Case B). Readings were recorded at every 2° for both *θ* and *ϕ* orientation.

For validation, two measurements were carried out; return loss (*S_11_*) and radiation pattern measurement. Case A and Case B conditions were tested for both measurements, as illustrated in Figure 9a,b, respectively. The *S_11_* which indicates the impedance matching performance of an antenna was measured by using VNA; meanwhile the measurement of the radiation pattern was conducted in an anechoic chamber. In 3D radiation pattern measurement, the antenna was rotated on a turn-table in theta (*θ*) and phi (*ϕ*) axis, and the radiated field was measured at each angle. Figure 10 shows the measurement configuration.

**Figure 11:**
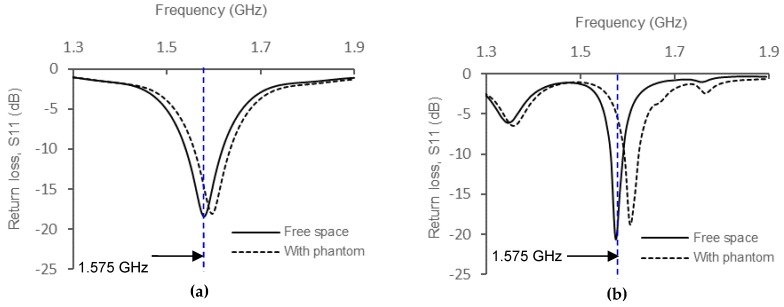
(**a**) Simulated return loss (*S_11_*) of the antenna; (**b**) Measured return loss (*S_11_*) of the antenna. Small deviations are observed for both cases, where the resonant frequency has shifted to the right due to the changes in input impedance (*Z_in_*) when the phantom is added.

Comparison of *S_11_* between measured and simulated results are shown in Figure 11. As predicted, the resonant frequency shifted slightly to the right when the antenna was tested in the presence of the muscle phantom, and the effect was significant during measurement. This effect is called detuning effect, and is calculated based on Equation (4):

(4) $$\mathrm{Detuning}\;\%=\frac{Detuning\Delta }{f_{o}}\times 100$$

**Figure 12:**
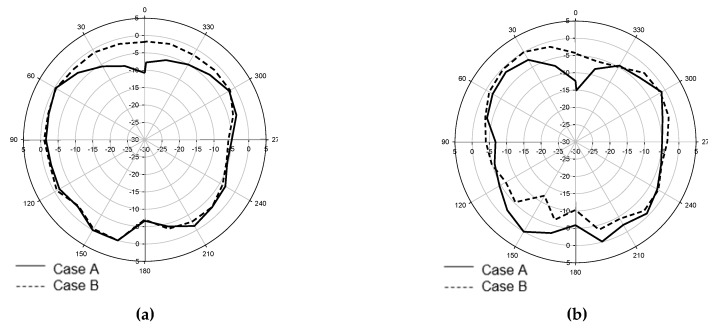
Measured radiation pattern: (**a**) Electric field plane (E-plane); (**b**) Magnetic field plane (H-plane). Slight deformation is observed due to the loss in radiated power that is absorbed by the lossy medium.

By comparing the amount of frequency detuning to the desired resonant frequency through simulation results, the phantom with the lowest *ε_r_* and *σ*, which is body fat has the smallest detuning percentage (1.14%) as compared to the phantoms with higher *ε_r_* and *σ* (e.g., muscle and blood) which give 1.4% each. This effect is due to the changes in the antenna’s internal impedance, *Z_in_* when it was placed on a material having high loss (tan*δ*) and significant *σ* value. The correlation between the presence of lossy dielectric materials for various *ε_r_* to *Z_in_* will be discussed in the next section. Figure 12 shows the radiation pattern of the e-textile antenna for both cases in E-plane and H-plane, respectively. Based on the graphs, slight deformation in radiation pattern was observed which indicates that in terms of radiation behavior, the on-body condition did not give much influence as compared to the free space environment. However, based on the efficiency data, significant losses were observed due to the reflection of signal or impedance mismatch, which will be clarified through near-field observation in the next section.

## 6. Discussion and Analysis

Electromagnetic radiation of the antenna was expected to be affected by the human body and may cause alteration in the antenna’s performance. Furthermore, human flesh has high *σ* and different dielectric properties as compared to a free space environment, thus this might have influenced its performance. Through simulation and measurement, the antenna’s *Z_in_* significantly changed when it was in close proximity with the human body. This section presents the analysis on the effects of the phantom’s properties e.g., *ε_r_* to overall antenna performance, through investigation of the simulated near-field distribution. The near-field computations were conducted to observe the electrical characteristics of each case which have affected performance such as impedance and efficiencies.

### 6.1. Correlation Between On-Body Condition with Input Impedance, Z_in_

**Figure 13:**
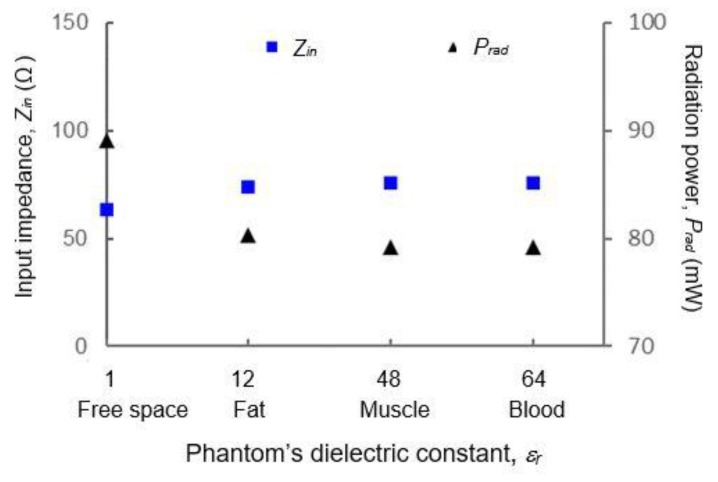
Input impedance and radiated power of the antenna when located near phantoms of various *ε_r_*. It shows that *Z_in_* is highly affected by the lossy medium, which has resulted in degradation of efficiency due to lower *P_rad_.*

Input impedance *Z_in_* is an important parameter because it determines how well the power is radiated from the antenna (described by radiation resistance, *R_r_*) or removed as heat (due to losses). When the dielectric material is not lossless, some power will be converted as heat, defined by load resistance, *R_L_*. Reactance, *X* on the other hand is related to reactive near-field that oscillates near the antenna. Figure 13 shows the changes in *Z_in_* when *ε_r_* is varied from 1 (free space) to 64 (human blood), calculated based on both resistance (*R_r_*, *R_L_*) and reactance (*X*). Based on this diagram, the value of *ε_r_* only does not justify precisely (in magnitude) the amount of changes in *Z_in_* because in these cases, phantoms with higher *ε_r_* such as blood has a higher tan*δ* due to higher conductivity (*σ* = 1.9 S/m) as compared to phantoms with lower *ε_r_* such as body fat (*σ* = 0.17 S/m). Hence, more than one parameters are varied. However, it can be observed that the presence of lossy dielectric on the antenna has made significant changes to its *Z_in_*, mainly due to the increase in *R_L_* and *X*. For example, when the antenna is put on top of the body fat phantom, there is an increase of 17% in *Z_in_*, followed by 19% for muscle and 20% for blood. The increase of *Z_in_* is also expected due to the fringing effect that has significantly taken place when a phantom was added, which resulted in the changes of electrical size of the antenna’s radiating element. The phantom added capacitive effect to the antenna thus, the capacitive reactance, *X_c_* was higher and the resonant frequency was shifted to a higher value. Due to the impedance change, mismatch occurred, which resulted in degradation of radiated power, *P_rad_* by the antenna as shown by the graph where the *P_rad_* is reduced by 9% for fat, and 11% for muscle and blood. This was proven through efficiency performance of the antenna which has reduced significantly especially when a phantom with a higher *ε_r_* was added.

### 6.2. Correlation Between On-Body Condition with Near-Field Distribution

**Figure 14:**
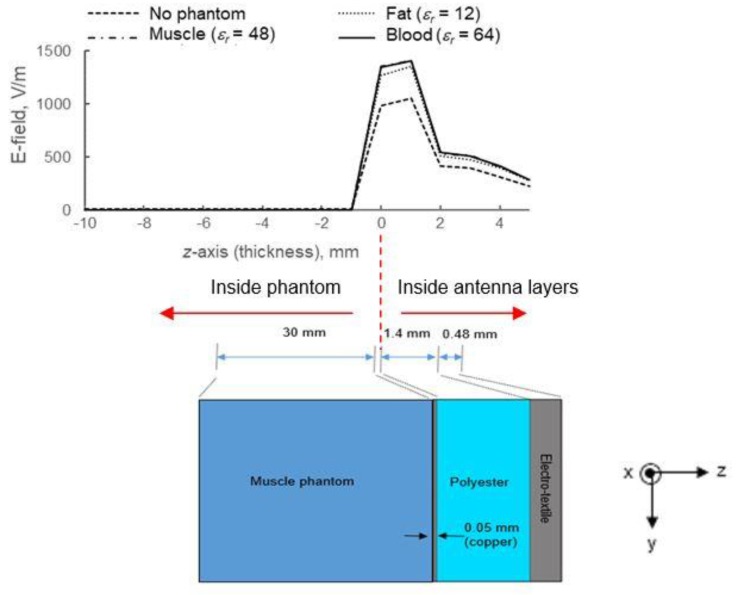
Graph of electric field intensities along antenna and phantom layers (*z*-axis), which shows the difference in E-field magnitude on the antenna surface for various *ε_r_*. As compared to the free space (no phantom) condition, the amount of E-field intensity (V/m) inside the antenna layer is higher in the presence of a phantom, which is expected due to the increase in *Z_in_* as observed in Figure 13.

The previous section has shown that antenna performance is highly affected when it is located very near to the human body (phantom). According to [18], detuning effects will be higher when the separation distance between an antenna and an object is lower due to significant alteration to its near-field distribution caused by the object. In this paper, the separation distance is almost zero. Therefore, in order to assess this situation, the electric and magnetic field’s strengths in the reactive near-field region are analyzed in this section. The reactive near-field region, *R_NF_* is defined as *R_NF_* $\leq 0.62\sqrt{D^{3}/\lambda }$, where *D* is the aperture size of the antenna. Figure 14 shows the electric field behavior along the *z*-axis of antenna and phantom layers as described in Figure 7. There is a significant amount of fields propagating inside antenna layers when a phantom is attached to the antenna. Due to high loss characteristics in the phantom, some power was absorbed through the dielectric layers of the antenna which reduced its efficiency. 

**Figure 15:**
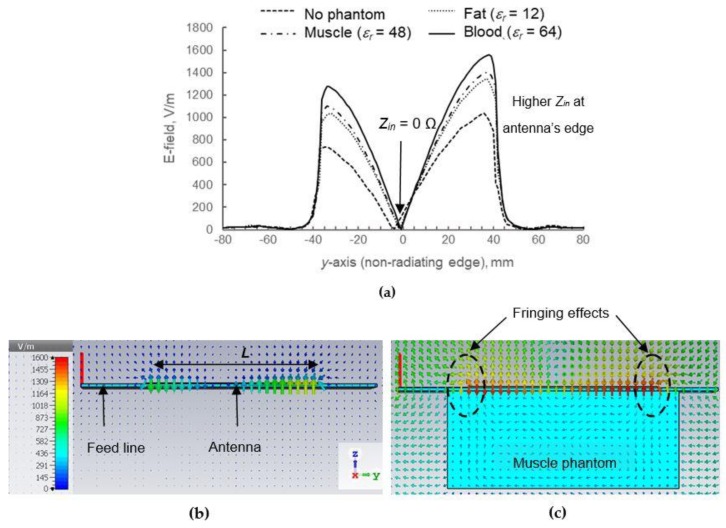
Near-field electric field intensity: (**a**) Distribution graph along *y*-axis (non-radiating edge); (**b**) 3D view (without phantom); (**c**) 3D view (with muscle phantom, *ε_r_* = 48).

Figure 15a illustrates the electric field (E-field) distribution along non-radiating edges, *L* of the antenna. The antenna’s radiating element behaves as a perfect electric conductor at the surface; therefore, based on the graph, the electric-field density is zero at the patch surface. Towards the edge, the E-field is increasing due to the rise of *Z_in_*. By comparing the cases when a phantom is added, the E-field increases with respect to the *ε_r_* and *σ*. For example, the maximum E-field increased to 29.7% in fat, followed by 35.3% in muscle and 36.1% in blood. Based on theoretical current-voltage relation, this E-field observation correlates to the increment of impedance, *Z_in_* as explained in Figure 13, which indicates that more energy is trapped inside the antenna layer when a phantom is added instead of radiated to the air. Figure 15b,c show the E-field distribution in two-dimensional views, showing higher magnitude when a phantom is added. The fringing fields in Figure 15c are clearly observed, which are predicted to be one of the factors in performance degradation. The increase in E-field intensity is also observed from the colors of arrows in this diagram.

**Figure 16:**
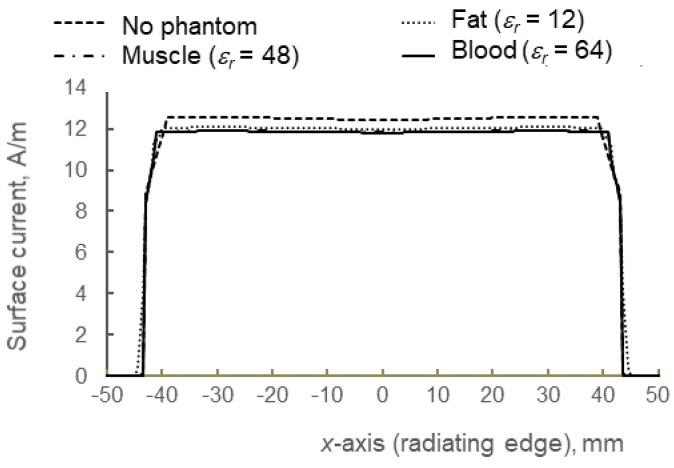
Magnetic surface currents along the *x*-axis (radiating edge). As the values of surface currents are similar for all cases, this factor can be omitted. It can be observed that when surface current is constant, high E-field intensity (V/m) for higher *ε_r_* and *σ* case is mainly due to the increase in *Z_in_* in lossy medium.

**Figure 17:**
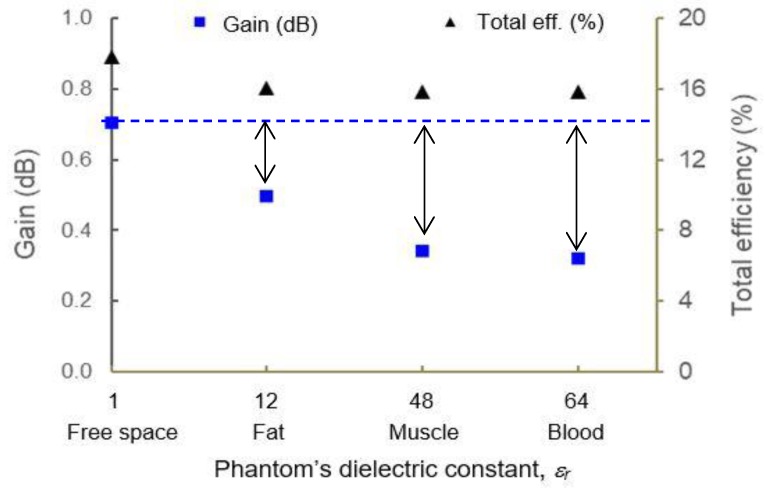
Efficiency and gain of e-textile antenna for various phantom properties. From an ideal antenna perspective, the efficiency is very low, which is due to the low conductivity and thickness of the developed material; however, through observation on the trend, it is clear that higher loss medium will result in lower gain.

Figure 16 shows the magnetic surface currents along radiating edges, *W* of the antenna. The currents are almost uniformly distributed throughout the antenna surface, with an average reading of 12.1 A/m and a standard deviation of only 0.27. It is important to ensure the constant behavior of current in order to validate the correlation between E-field intensity and *Z_in_* as previously explained through current-voltage relation. Based on comparison to electric field magnitude shown in Figure 14 and Figure 15, there is a clear correlation in how the E-fields are responsible for radiation performance, as illustrated by gain and total efficiency in Figure 17. As the phantom becomes more lossy (from fat to blood), the total efficiency has dropped by −1.74% (in fat) and −2% (in blood) in comparison to free space performance, and thus the gain has also decreased e.g., −0.21 dB (fat), followed by −0.37 dB (muscle) and −0.39 dB (blood). Further design optimizations are needed in this antenna to increase the gain and efficiency, such as by increasing the *σ* of the e-textile material (currently, *σ* = ~10^4^ S/m is considered as low); however for a comparison study, clear observation on the factors that may affect the antenna performance in free space and on-body has been achieved through detailed analysis of near-field computation. Table 3 shows the detailed performance deviation of the antenna when located near a human phantom. 

### 6.3. Scope and Limitations

The scope of the project is limited to on-body investigation, the main focus is to provide detailed investigation on how the antenna performance and behavior are changed with respect to the phantom’s parameters. In the context of wearable technology, the antenna will be located very close to the human body, thus a detailed study on the field strength at reactive near-field region is very important. For validation, human muscle which makes up the largest proportion of the human arm was chosen for fabrication and testing. The shape of the muscle phantom was simplified to a cuboid shape and the size was about the same as the size of the antenna’s radiator. All experiments in this paper were not conducted with actual human participants to ensure consistency of test environment and to reduce other uncertainties such as human body movement. Furthermore, during far-field radiation pattern measurement, high power electromagnetic waves were transmitted to the antenna-under-test at a very close range, thus by using a human phantom, safety and health precautions were ensured. Near-field simulation results in this paper were used to analyze and validate the behavior of the antenna in two conditions; free space and on-body. Actual near-field measurement was not conducted due to the unavailability of a near-field probe which is very costly and requires expensive chamber setup (~millions in USD). Other conditions such as bending or stretching are not included in this paper to maintain the consistency of the discussion and analysis. 

In the future, based on the analysis presented in this paper, optimization works can be done to improve the performance of the antenna in both free space and on-body conditions. It is clearly observed that the task of designing antennas located near human body parts with different properties (*ε_r_*, *σ*, tan*δ*) requires more considerations as compared to free space. Due to the complexity of the environment that can significantly affect the antenna’s performance, more optimization works are needed. One solution is by designing the antenna with *Z_in_* that is lower than the system impedance so that mismatch loss can be minimized in on-body condition. A high bandwidth antenna can also be designed to reduce frequency detuning effects. Other than that, based on the data shown in this paper, the usage of a ‘buffer-like layer’ can also be proposed. 

## 7. Integration with GPS Tracking Device

**Figure 18:**
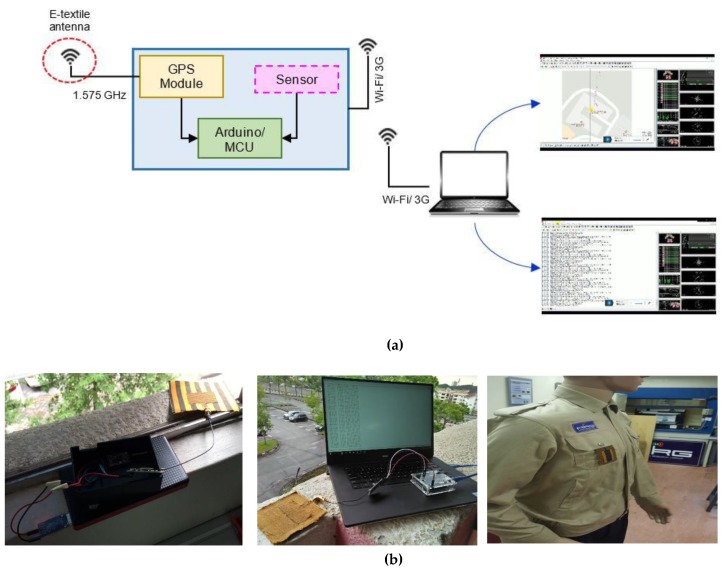
(**a**) Block diagram of GPS tracking system test bench; (**b**) Measurement setup of the integration of e-textile antenna with GPS tracking module. During operation, precise and consistent real time GPS data (location) were received at the server (computer) without any losses or intermittence.

In order to verify the ability of the antenna for tracking application, the most optimum antenna prototype (despite the performance change) was incorporated and tested in a GPS tracking system test bench, as illustrated in the basic block diagram in Figure 18. To demonstrate the receiving capability, a GPS module was integrated with the e-textile antenna operating at 1.575 GHz and connected to Arduino board that was attached to a Wi-Fi/3G antenna for data transmission to the server. From the server, the precise location of the e-textile antenna was displayed, as shown in Figure 18a. Based on the data received at the computer, the GPS wearable antenna developed in this research worked well in receiving GPS data with no data loss within the required time interval, which indicates that the technical performance of the antenna in terms of its resonant frequency, impedance and gain was sufficient to act as an important sub-system to the GPS tracking device. 

## 8. Conclusions

The correlation between a phantom’s dielectric properties to antenna technical performance such as impedance, electric field and gain was analyzed systematically in this paper. As validated through simulation and measurement, the presence of a phantom added some capacitive elements in the antenna structure, thus resulted in the shift of resonant frequency due to higher capacitive reactance in the input impedance value. Investigation through near-field distribution has been done, which agrees very well with the theoretical current-voltage relation that shows that as the impedance is increasing, mismatch occurs. Thus, at 1.575 GHz, the electric field intensity inside the antenna is increased and less power is radiated by the antenna to its surroundings, which can affect the key performance of the antenna such as gain and efficiency. The flow of the study and parameters evaluated in this paper can be established as good methods in analyzing and validating the reason for performance degradation when an antenna is located near the human body. These findings are very useful to antenna designers, especially in determining the best location for their wearable antennas. 

## Figures and Tables

**Table 1:** The physical and electrical properties of fabricated e-textile.

Properties	Values
Conductivity (*σ*)	2.2 × 10^4^ S/m
Thickness (*t*)	0.48 mm
Fabric composition	83% Copper + 17% Polyester

**Table 2:** Chemical composition of the human muscle phantom.

Ingredients	Purpose	Quantity
Deionized water	Main component	420 mL
Agar	Polyethylene powder	20 g
Sodium dehydroacetate	As a preservative	0.25 g
Xanthan gum	As a thickener	6.25 g
Sodium chloride (NaCl)	To control the *σ* of phantom	4.1 g
Polyethylene powder	To control the *ε_r_* of phantom	29 g

**Table 3:** Changes in the antenna’s performance variables when located on a human phantom in comparison to a free space condition. The significance of each parameter is explained in the paragraph.

Condition	Fat (*ε_r_* = 12)	Muscle (*ε_r_* = 48)	Blood (*ε_r_* = 64)
Detuning %	1.14	1.4	1.4
*ΔZ_in_* (%)	17 ↑	19 ↑	20 ↑
*ΔP_rad_* (%)	9 ↓	11 ↓	11 ↓
*Δ*E-field intensity (%)	29.7 ↑	35.3 ↑	36.1 ↑
*Δ*Total efficiency %	1.74 ↓	2 ↓	2 ↓
*Δ*Gain (dB)	0.21 ↓	0.37 ↓	0.39 ↓

Note: ↑ indicates positive change in the variables and ↓ indicates negative change in the variables.
